# bFGF-like Activity Supported Tissue Regeneration, Modulated Neuroinflammation, and Rebalanced Ca^2+^ Homeostasis following Spinal Cord Injury

**DOI:** 10.3390/ijms241914654

**Published:** 2023-09-27

**Authors:** Alessio Ardizzone, Valentina Bova, Giovanna Casili, Alessia Filippone, Marika Lanza, Alberto Repici, Emanuela Esposito, Irene Paterniti

**Affiliations:** Department of Chemical, Biological, Pharmaceutical and Environmental Sciences, University of Messina, Viale Ferdinando Stagno D’Alcontres, 31, 98166 Messina, Italy; aleardizzone@unime.it (A.A.); valentina.bova@unime.it (V.B.); gcasili@unime.it (G.C.); afilippone@unime.it (A.F.); mlanza@unime.it (M.L.); alberto.repici@unime.it (A.R.); ipaterniti@unime.it (I.P.)

**Keywords:** spinal cord injury (SCI), basic fibroblast growth factor (bFGF), SUN11602, neuroinflammation, Ca^2+^ homeostasis, neurotrophic factors

## Abstract

A spinal cord injury (SCI) is a well-defined debilitating traumatic event to the spinal cord that usually triggers permanent changes in motor, sensory, and autonomic functions. Injured tissue becomes susceptible to secondary mechanisms caused by SCIs, which include pro-inflammatory cytokine release, the activation of astrocytes and microglia, and increased neuronal sensibility. As a consequence, the production of factors such as GFAP, IBA-1, TNF-α, IL-1β, IFN-γ, and S100-β slow down or inhibit central nervous system (CNS) regeneration. In this regard, a thorough understanding of the mechanisms regulating the CNS, and specifically SCI, is essential for the development of new therapeutic strategies. It has been demonstrated that basic fibroblast growth factor (bFGF) was successful in the modulation of neurotrophic activity, also promoting neurite survival and tissue repair, thus resulting in the valuable care of CNS disorders. However, bFGF therapeutic use is limited due to the undesirable effects developed following its administration. Therefore, the synthetic compound mimetic of bFGF, SUN11602 (with chemical name 4-[[4-[[2-[(4-Amino-2,3,5,6-tetramethylphenyl)amino]acetyl]methylamino]-1-piperidinyl]methyl]benzamide), has been reported to show neuroprotective activities similar to those of bFGF, also demonstrating a good pharmacokinetic profile. Here, we aimed to investigate the neuroprotective activity of this bFGF-like compound in modulating tissue regeneration, neuroinflammation, and Ca^2+^ overload by using a subacute mouse model of SCI. SUN11602 (1, 2.5, and 5 mg/kg) was administered orally to mice for 72 h daily following the in vivo model of SCI, which was generated by the extradural compression of the spinal cord. The data obtained demonstrated that SUN11602 treatment considerably decreased motor alteration and diminished the neuroinflammatory state through the regulation of glial activation, the NF-κB pathway, and kinases. Additionally, by controlling Ca^2+^-binding proteins and restoring neurotrophin expression, we showed that SUN11602 therapy restored the equilibrium of the neuronal circuit. Because of these findings, bFGF-like compounds may be an effective tool for reducing inflammation in SCI patients while enhancing their quality of life.

## 1. Introduction

A spinal cord injury (SCI) is a traumatic event that results in debilitating conditions for the patient, as it causes reduced mobility and sensitivity of the lower and/or upper limbs [1]. Although there is no accurate estimate of the global prevalence of SCIs, according to the World Health Organization (WHO), about 60 million people per year become affected, with up to 90% of these cases resulting from traumatizing events like falls and motor vehicle accidents [2].

Therefore, SCIs are considered one of the most common neurological insults worldwide, resulting in severe disability, which significantly impacts the quality of life in affected patients [3]. To date, the therapeutic tools to treat SCIs include surgery combined with a methylprednisolone sodium succinate treatment, which is a drug frequently administered after acute phases of spinal trauma [4]. Despite the fact that methylprednisolone sodium succinate is an agent capable of improving antioxidant defenses [5], it fails to ameliorate neurite sprouting, the remyelination of axons, and functional recovery [6]. Therefore, the development of novel pharmacological approaches for SCI patients is still considered an important goal for clinical practitioners.

It is well recognized that SCI causes neurological impairments due to both primary and secondary injuries [7]. A “Primary” injury is defined as the irreparable mechanical damage to the spinal cord tissue that takes place immediately after the impact, whereas the “secondary” injury continues for several days or months, leading to more serious complications over time [8]. Particularly, this secondary phase is characterized by the infiltration of leukocytes and activation of glial cells that worsen tissue damage by releasing proteases, lysosome enzymes, and pro-inflammatory species [9]. SCI-induced inflammation has the potential to aggravate traumatic injury by inhibiting the formation of scar tissue, favoring neuronal death, and altering the physiological balance of several endogenous factors [10,11].

Considering the fact that SCIs are one of the most difficult study areas because there are no effective therapies available, targeting neuroinflammation could effectively contribute to the enhancing of neuroregeneration by mitigating secondary injury cascades [12].

Basic fibroblast growth factor (bFGF; FGF-2) is a key player in the central nervous system (CNS), because it is present in the mature brain and is involved in various functions, such as regulating neuronal proliferation, promoting neurotrophic activity and neurite survival, and even healing tissue damage [13].

Moreover, a clinical trial reported that the intrinsic brain levels of bFGF are known to increase following focal cortical infarcts, suggesting that it also plays an important role in glial cell modulation during recovery [14].

The latest reports state that bFGF exerts neuroprotective activity through interaction with fibroblast growth factor receptors (FGFRs) and specifically with FGFR1, improving neuronal survival in different pathological conditions [15,16]. In CNS diseases, bFGF/FGFR1 activation counteracts microglial neuroinflammation, attenuating IκBα phosphorylation and NF-κB p65 translocation, as well as decreasing the levels of pro-inflammatory mediators [17].

Unfortunately, a clinical investigation employing native bFGF in acute stroke patients failed to demonstrate full safety due to the observation of various adverse effects [14]. In particular, results demonstrated that exogenous bFGF treatment causes fever and leukocytosis, decreases systolic and diastolic blood pressure, and causes hypokalemia, with increased white blood cells and neutrophil counts as well as blood glucose levels [14]. Because of this, it was necessary to synthesize bFGF functional analogues in order to create safer drugs. Hence, an aniline-derived chemical called SUN11602 was produced to overcome these limitations. This novel compound exposed peculiar pharmacokinetic qualities and a higher bioavailability in all the animal species examined, including rats, mice, and dogs [18].

Similar to bFGF, SUN11602 interacts with the FGFR1 receptor, increasing its phosphorylation and promoting neuronal survival and development in pathological conditions [19]. Additionally, the SUN11602-induced activation of FGFR1 enhances the production of the Calbindin-D28k protein (also known as Calbindin 1 or Calb1), which is extensively expressed in several brain regions [20].

Calbindin-D28k is a Ca^2+^ binding protein that acts as a calcium buffer and calcium sensor, thus becoming highly involved in maintaining Ca^2+^ intracellular homeostasis [20].

Previously, the remarkable pharmacological activities of SUN11602 have been demonstrated to decrease PD pathological hallmarks and neuroinflammation [18]. Considering the aforementioned beneficial effects, in this study, we aimed to evaluate the neuroprotective skills of SUN11602 in a subacute model of an SCI induced by extradural compression of the spinal cord, in order to investigate its effect on tissue regeneration as well as the management of neuroinflammatory signaling pathways.

## 2. Results

### 2.1. SUN11602 Treatment Counteracted Motor Deficits and Tissue Damage, and It Reduced the MPO Activity following SCI

**Figure 1:**
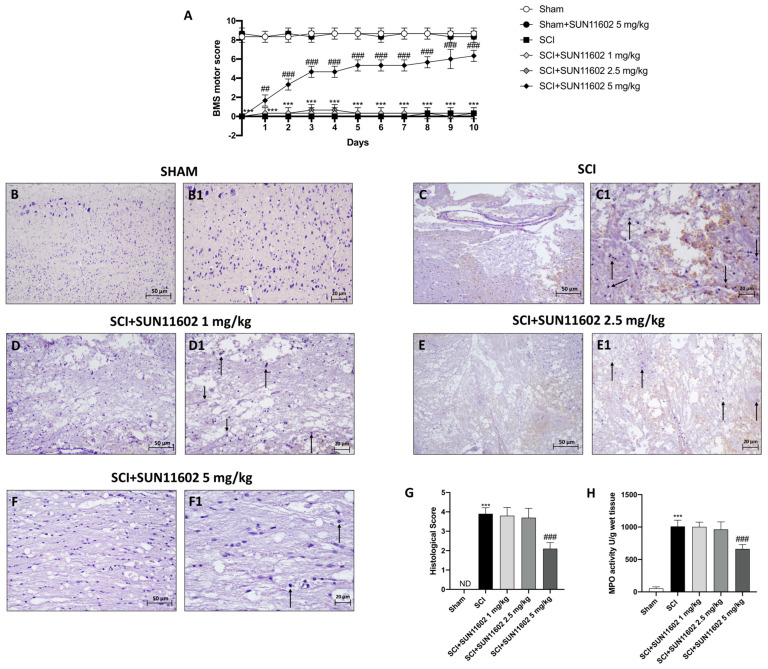
SUN11602 promoted motor recovery, improved spinal cord tissue damage, and downregulated MPO activity after an SCI. From the first day of treatment with SUN11602 at 5 mg/kg, the mice already showed lessened motor deficits compared with the SCI group (**A**). Histological evaluations indicated the loss of spinal cord morphology in the SCI group (**C**,**C1**,**G**) than in the Sham group (**B**,**B1**,**G**). SUN11602, especially at the dose of 5 mg/kg, restored morphological tissue (**F**,**F1**,**G**), whereas SUN11602 at the doses of 1 mg/kg (**D**,**D1**,**G**) and 2.5 mg/kg were not efficacious (**E**,**E1**,**G**). MPO activity levels were high in the SCI group compared with the control group (**H**). Only SUN11602 at 5 mg/kg reduced MPO levels (**H**). Black arrows indicate morphological changes. The results of the histological evaluations are displayed at 20× and 40× magnifications. Data are expressed as mean ± SD where N = 10 mice for each group. One–Way and Two-way ANOVA test. *** *p* < 0.001 vs. Sham; ^##^ *p* < 0.01 vs. SCI, ^###^ *p* < 0.001 vs. SCI; ND: not detectable.

One of the main consequences that occur following an SCI is the loss of neuromotor function. For this purpose, a BMS test was performed for 10 days to assess the effect of SUN11602 on motor function. Only SUN11602 at a dose of 5 mg/kg was able to significantly restore motor function after an SCI procedure, starting from the first day of administration (Figure 1A). By contrast, 1 mg/kg and 2.5 mg/kg doses of SUN11602 have not been shown to be effective in reducing motor deficits (Figure 1A).

In addition, we performed H&E analysis in order to evaluate tissue morphology following trauma. SCI+vehicle mice revealed an alteration in the morphological structure of the perilesional area (Figure 1C,C1, score Figure 1G) compared with the Sham group (Figure 1B,B1, score Figure 1G). The oral administration of SUN11602 at 5 mg/kg induced tissue restoration three days after the SCI (Figure 1F,F1, score Figure 1G). No protective effect was observed when SUN11602 was administered at the lower doses of 1 mg/kg (Figure 1D,D1, score Figure 1G) and 2.5 mg/kg (Figure 1E,E1, score Figure 1G).

Post-traumatic inflammation is also characterized by infiltrating cells, especially neutrophils [21]. In this regard, we performed the MPO assay as a specific indicator of the extent of neutrophil infiltration [21]. Our results showed that MPO levels were increased in the SCI group compared with the Sham group (Figure 1H). SUN11602 oral treatment, at a dose of 5 mg/kg, significantly reduced MPO activity levels (Figure 1H), whereas SUN11602 at 1 mg/kg and 2.5 mg/kg were not effective in diminishing MPO levels (Figure 1H).

### 2.2. SUN11602 Treatment Lessened Neuronal Demyelination following SCI

**Figure 2:**
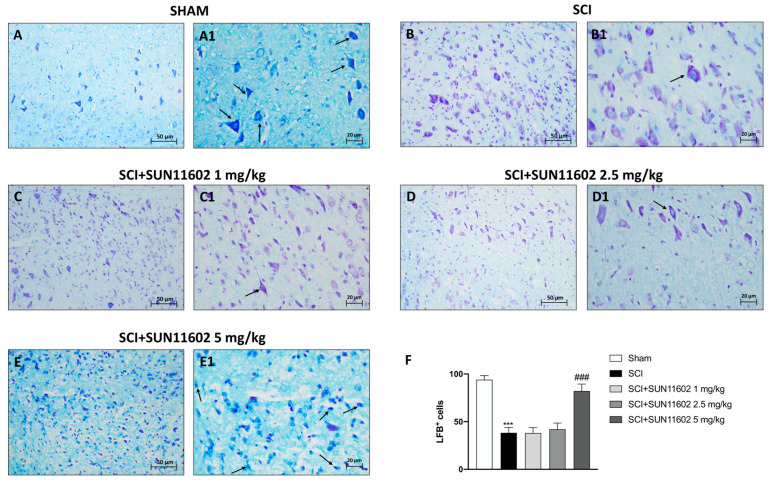
SUN11602 treatment restored neuron myelinization. The SCI group showed a high degree of the demyelination of neurons (**B**,**B1**,**F**) compared with the Sham group (**A**,**A1**,**F**). The oral treatment of SUN11602 at 5 mg/kg meaningfully restored the myelinization of the neurons three days after SCI (**E**,**E1**,**F**). Administrations of SUN11602 at 1 mg/kg (**C**,**C1**,**F**) and 2.5 mg/kg (**D**,**D1**,**F**) were not effective in counteracting neuronal demyelination. Black arrows indicate myelinization. Images are shown at 20× and 40× magnifications. Data are expressed as mean ± SD where N = 10 mice for each group. One–Way ANOVA test. *** *p* < 0.001 vs. Sham; ^###^ *p* < 0.001 vs. SCI.

The inflammatory response induced by an SCI causes the axonal degradation of myelinated fibers, thus producing myelin debris and vacuoles in the white matter of the spinal cord. In light of this, we examined the number of LFB-positive neurons by employing Luxol Fast Blue (LFB) staining. The SCI group showed a significantly reduced number of LFB^+^ neurons (Figure 2B,B1, score Figure 2F) compared with the Sham group (Figure 2A,A1, score Figure 2F). Oral treatment with SUN11602 at 5 mg/kg showed a remarkable restoration of neuron myelination (Figure 2E,E1, score Figure 2F). On the contrary, LFB staining did not give positive results following SUN11602 administration at doses of 1 mg/kg (Figure 2C,C1, score Figure 2F) and 2.5 mg/kg (Figure 2D,D1, score Figure 2F).

### 2.3. SUN11602 Treatment Reduced Mast Cell Infiltration following an SCI

**Figure 3:**
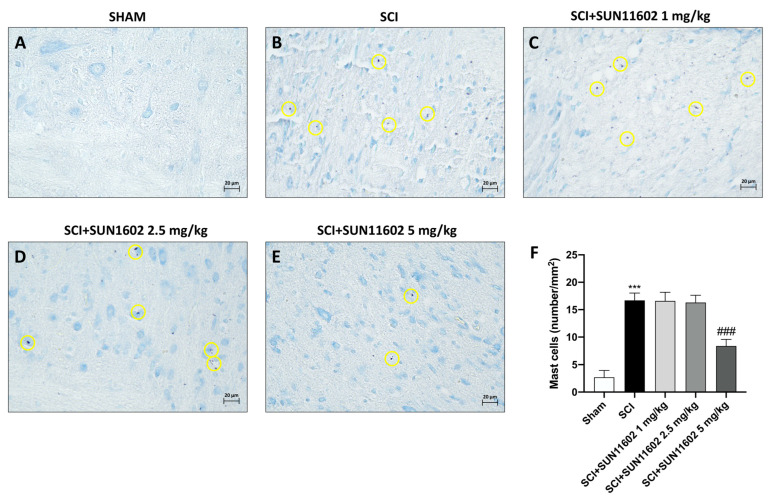
SUN11602 reduced mast cell infiltration in spinal cord tissue. The SCI caused the recruitment of mast cells in the damage site (**B**,**F**) than in the control group (**A**,**F**). Only at a dose of 5 mg/kg was SUN11602 capable of reducing the number of mast cells (**E**,**F**), in comparison with SUN11602 at 1 mg/kg (**C**,**F**) or 2.5 mg/kg (**D**,**F**) doses, which were ineffective. Yellow circles indicate the mast cells. Images are displayed at 40× magnifications. Data are expressed as mean ± SD where N = 10 mice for each group. One–Way ANOVA test. *** *p* < 0.001 vs. Sham; ^###^ *p* < 0.001 vs. SCI.

Following trauma, we identified mast cells in the perilesional area of the spinal cord by using toluidine blue staining. In this manner, an elevated presence of mast cells was observed in spinal cord tissue three days after an SCI (Figure 3B, score Figure 3F) compared with the control group (Figure 3A, score Figure 3F). SUN11602 oral administration at a dose of 5 mg/kg significantly reduced the number of mast cells (Figure 3E, score Figure 3F). SUN11602, at the doses of 1 mg/kg (Figure 3C, score Figure 3F) and 2.5 mg/kg (Figure 3D, score Figure 3F), did not demonstrate a significant reduction in mast cell numbers after an SCI. The results collected so far have highlighted the ineffectiveness of doses 1 mg/kg and 2.5 mg/kg of SUN11602. Therefore, we decided to continue the following analysis by only examining SUN11602 at a 5 mg/kg dose.

### 2.4. The Effect of SUN11602 Treatment on Glial Cell Activation following an SCI

**Figure 4:**
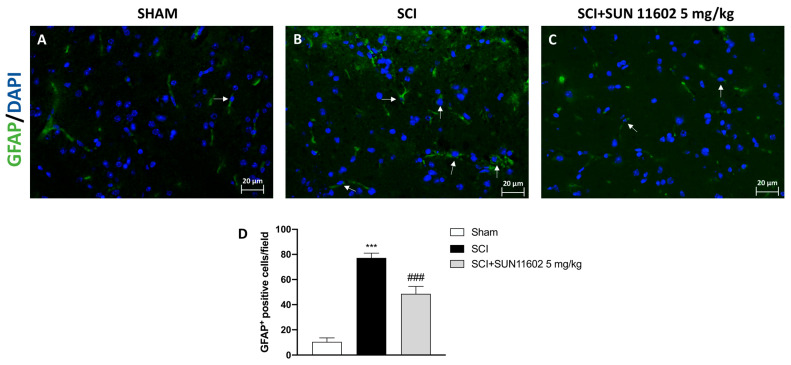
SUN11602 reduced the number of GFAP^+^ cells in SCI mice. SCI mice showed positive staining for GFAP (**B**,**D**) in comparison with the control group (**A**,**D**). The mice that received the SUN11602 5 mg/kg treatment showed a marked decrease in GFAP positive cells (**C**,**D**). White arrows indicate positive cells. Images are displayed at 40× magnifications. Data are expressed as mean ± SD where N = 10 mice for each group. One–Way ANOVA test. *** *p* < 0.001 vs. Sham; ^###^ *p* < 0.001 vs. SCI.

**Figure 5:**
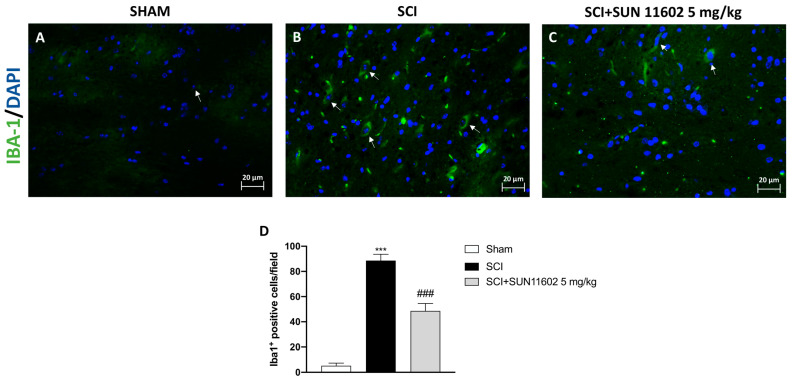
SUN11602 reduced the number of IBA-1^+^ cells in SCI mice. Spinal cord tissues from SCI mice revealed a high number of IBA-1 positive cells (**B**,**D**), compared with the Sham group (**A**,**D**). IBA-1 expression was decreased after the SUN11602 5 mg/kg oral treatment (**C**,**D**). White arrows indicate positive cells. Images are displayed at 40× magnifications. Data are expressed as mean ± SD where N = 10 mice for each group. One–Way ANOVA test. *** *p* < 0.001 vs. Sham; ^###^ *p* < 0.001 vs. SCI.

Through immunofluorescence staining, we detected the number of cells expressing GFAP and IBA-1 as reliable markers of reactive astrocytes and microglia after an SCI. The control group showed a low number of positive cells for both GFAP and IBA-1 (Figure 4A and Figure 5A, scores Figure 4D and Figure 5D, respectively), whereas a high number of positive cells for GFAP and IBA-1 was found in the SCI group (Figure 4B and Figure 5B, scores Figure 4D and Figure 5D, respectively). Then, three days after the SCI, the SUN11602 5 mg/kg oral treatment considerably reduced GFAP and IBA-1 positive cells (Figure 4C and Figure 5C, scores Figure 4D and Figure 5D, respectively).

### 2.5. SUN11602 Treatment Exerted an Anti-Inflammatory Effect by Modulating NF-κB and Its Interplay with PI3K/AKT Axis and p38 MAPK following an SCI

**Figure 6:**
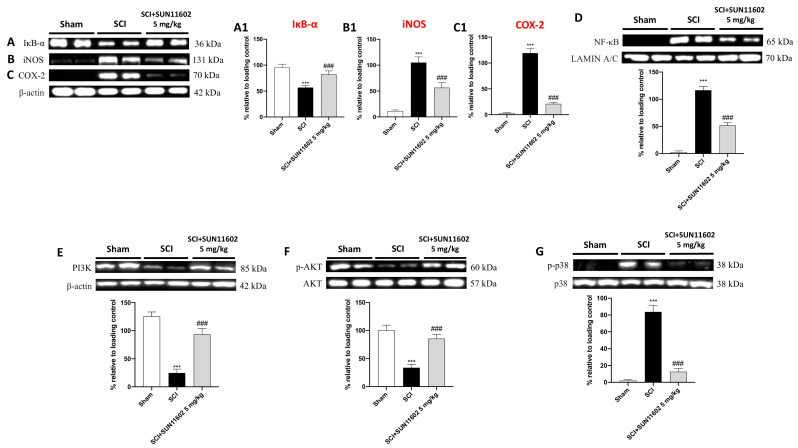
SUN11602 modulated NF-κB pathway, PI3K/AKT axis, and p38 MAPK following an SCI. Western blot analysis showed NF-κB pathway activation in SCI mice compared with the control group (**A**,**A1**,**D**). The SUN11602 5 mg/kg oral treatment successfully modulated the NF-κB pathway by restoring IκB-α while diminishing NF-κB expression (**A**,**D**). Pro-inflammatory markers such as iNOS and COX-2 were highly expressed in the SCI group (**B**,**B1**,**C**,**C1**). The SUN11602 5 mg/kg administration reduced the expression of both inflammatory markers (**B**,**B1**,**C**,**C1**). The SCI group showed low levels of expression of PI3K/Akt compared with the Sham group (**E**,**F**). The SUN11602 5 mg/kg oral treatment restored the PI3K/Akt pathway (**E**,**F**). P-p38 levels were increased in the SCI group than in the control group; SUN11602 at 5 mg/kg was able to reduce its expression (**G**). Data are expressed as mean ± SD where N = 10 mice for each group. One–Way ANOVA test (*p* < 0.05) followed by Bonferroni post hoc test for multiple comparisons; *** *p* < 0.001 vs. Sham; ^###^ *p* < 0.001 vs. SCI.

In response to traumatic events like an SCI, several proinflammatory species are released in the perilesional region. These mediators increase local inflammation and worsen the consequences of SCI secondary damage. Thus, using western blot analysis, we probed SUN11602′s anti-inflammatory activity by evaluating its action on the NF-κB pathway, one of the main orchestrators of the inflammatory course. SCI mice presented a low expression of IκB-α compared with the control group (Figure 6A, score Figure 6A1). The SUN11602 5 mg/kg oral treatment restored IκB-α levels almost to baseline values (Figure 6A, score Figure 6A1). The trauma inflammatory response determined an upregulation of NF-κB in the SCI group compared with the control group (Figure 6D). The SUN11602 at 5 mg/kg treatment considerably reduced translocation into the nucleus of NF-κB (Figure 6D). Moreover, to confirm the capability of SUN11602 of modulating inflammatory responses, we assessed COX-2 and iNOS expression through western blot analysis. Both pro-inflammatory markers were highly expressed in the SCI group compared with the control group (Figure 6B,C, score Figure 6B1,C1). SUN11602 5 mg/kg treatment substantially decreased iNOS and COX-2 levels (Figure 6B,C, score Figure 6B1,C1), suggesting a notable anti-inflammatory effect.

Another important signaling pathway in an SCI is represented by PI3K/AKT axis [22].

Following an SCI procedure, the mice revealed a considerable downregulation of PI3K/AKT compared with the basal levels of the Sham group (Figure 6E,F, respectively). Oral treatment with SUN11602 at 5 mg/kg was capable of restoring their expression (Figure 6E,F, respectively). Moreover, the p38 protein, belonging to the mitogen-activated protein kinase (MAPK) family, is activated by various external stimuli, including SCI-induced trauma, which plays an important role in the inflammatory response. In this regard, the SCI group showed an increase in the expression of the phosphorylated form of p38 (p-p38) compared with the Sham group (Figure 6G). By contrast, the oral administration of SUN11602 at 5 mg/kg notably decreased p-p38 expression (Figure 6G).

### 2.6. SUN11602 Administration Successfully Restored Ca^2+^-Homeostasis following an SCI

Many papers report a close correlation between neuroinflammation and Ca^2+^ dyshomeostasis [18,23]. In this context, it was demonstrated that bFGF maintains Ca^2+^ homeostasis and efflux via PKCδ activation [24]. Hence, since SUN11602 is a bFGF mimetic, we evaluated its capacity to regulate calcium-binding proteins such as Calbindin-D28K, S100-β, and Calpain by employing the western blot method.

**Figure 7:**
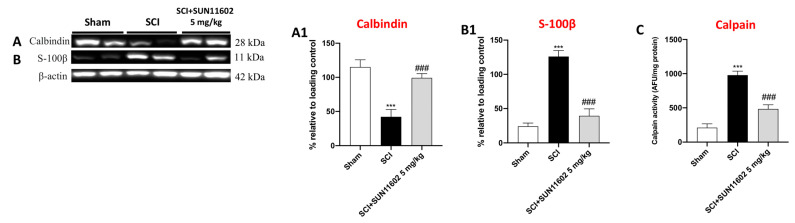
The modulation of Calcium homeostasis following SUN11602 treatment. The SCI group showed an important decrease in Calbindin expression compared with the Sham group (**A**), while SUN11602 induced a significant upregulation of Calbindin (**A**,**A1**). S-100β and Calpain levels were increased in mice subjected to SCIs compared with the Sham group (**B**,**B1**,**C**). However, the SUN11602 5 mg/kg oral administration was able to reduce their levels (**B**,**C**). Data are expressed as mean ± SD where N = 10 mice for each group. One–Way ANOVA test. *** *p* < 0.001 vs. Sham; ^###^ *p* < 0.001 vs. SCI.

A low expression of Calbindin-D28K was detected in the SCI+vehicle group compared with the Sham mice (Figure 7A, score Figure 7A1). The SUN11602 5 mg/kg oral administration considerably restored Ca^2+^ homeostasis, upregulating Calbindin-D28K expression (Figure 7A, score Figure 7A1). Conversely, SCI-induced trauma resulted in the upregulation of S100-β than in the control group (Figure 7B, score Figure 7B1). The SUN11602 5 mg/kg administration significantly downregulated S100-β expression three days after SCI (Figure 7B, score Figure 7B1). In addition, to further confirm SUN11602′s capability to regulate Ca^2+^ homeostasis through its binding proteins, an ELISA kit for Calpain was performed. The lack of Ca^2+^ homeostasis after SCI resulted in the activation of Calpain compared with the physiological levels of the Sham group (Figure 7C). Treatment with SUN11602 at 5 mg/kg was able to reduce Calpain levels (Figure 7C), suggesting a neuroprotective effect following an SCI, through the modulation of Ca^2+^ overload.

### 2.7. SUN11602 Treatment Modulated Neurotrophic Factor Levels following an SCI through CREB Induction

**Figure 8:**
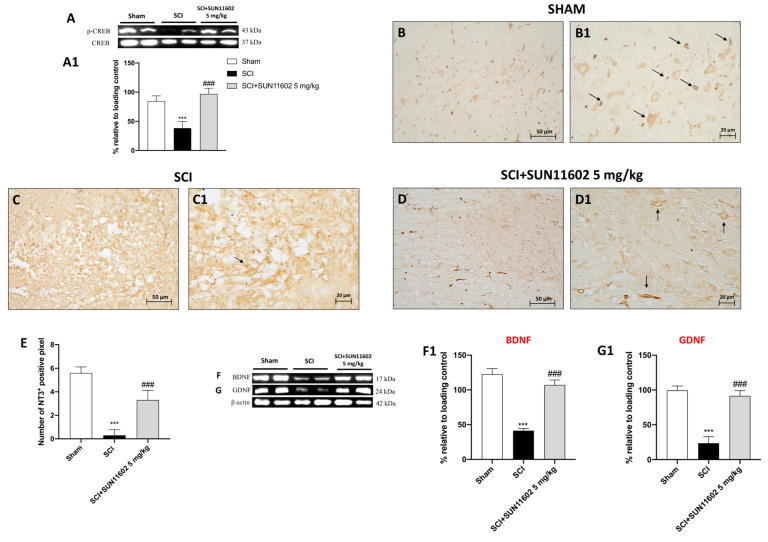
The SUN11602 administration restored the circuit of p-CREB/neurotrophins after an SCI. p-CREB expression was increased by SUN11602 at a 5 mg/kg administration following an SCI (**A,A1**). Immunohistochemical analysis showed NT-3 low positive staining in the SCI group (**C**,**C1**,**E**) compared with the Sham group (**B**,**B1**,**E**). The SUN11602 5 mg/kg treatment considerably increased NT-3 positive staining (**D**,**D1**,**E**). Contrarily, western blot analysis reported that BDNF and GDNF were reduced in mice subjected to SCIs (**F**,**F1**,**G**,**G1**). The oral administration of SUN11602 at 5 mg/kg determined an increase in BDNF and GDNF expression levels (**F**,**F1**,**G**,**G1**). Black arrows indicate positive cells, The results of the immunohistochemistry are presented at 20× and 40× magnifications. Data are expressed as mean ± SD where N = 10 mice for each group. One–Way ANOVA test. *** *p* < 0.001 vs. Sham; ^###^ *p* < 0.001 vs. SCI.

To further understand the regional changes in the expression of activated MAPK at the site of injury, we detected the phosphorylated form of transcription factor cyclic AMP-responsive element binding protein (p-CREB) expression as an important factor in upregulating endogenous neurotrophins. The SUN11602 5 mg/kg treatment determined an increase in p-CREB levels (Figure 8A, score Figure 8A1), suggesting that SUN11602 was capable of increasing its phosphorylation three days after an SCI. Thereafter, looking at neurotrophic factor expression, we found a low positive staining for NT-3 in the SCI group (Figure 8C,C1, score Figure 8E) compared with the Sham group (Figure 8B,B1, score Figure 8E). However, the oral treatment with SUN11602 at 5mg/kg notably restored NT-3 expression (Figure 8D,D1, score Figure 8E).

Moreover, to confirm the effect of SUN11602 on the p-CREB/neurotrophin circuit, BDNF and GDNF expression were evaluated using western blot analyses. Mice subjected to SCIs showed low BDNF and GDNF expression compared with the Sham group, in which their expression levels were basal (Figure 8F,G; scores Figure 8F1 and Figure 8G1, respectively). The SUN11602 5 mg/kg oral administration significantly restored BDNF and GDNF almost to the initial condition (Figure 8F,G; scores Figure 8F1 and Figure 8G1, respectively).

## 3. Discussion

SUN11602 is a synthetic compound capable of mimicking the neuroprotective activities of bFGF [25]. 

Although the biological activity of SUN11602 is still under investigation, its ability to prevent neuronal death while increasing the gene expression of CALB1 in cerebrocortical neurons in the cultures of the cerebrovascular neurons of rats has been demonstrated [25].

Moreover, Murayama et al. also demonstrated that SUN11602 increased the levels of Calbindin in neurons in mice by suppressing the increase in intracellular calcium due to glutamate excitotoxicity [25] and also in other clinical settings like Alzheimer’s disease [19].

Even though SUN11602 was developed as a bFGF mimic after chemical–pharmaceutical investigations, there are still specific differences that need to be noted.

Firstly, unlike bFGF, SUN11602 can either directly or indirectly trigger the phosphorylation of the cytosolic domain of the FGFR without binding to the extracellular domain of the FGFR-1 [25]. In addition, and in contrast to bFGF, SUN11602 shows no somatic cell proliferation [25].

Thus, considering SUN11602 a promising neuroprotective compound for many CNS disorders, this study investigated its bFGF-like activity in controlling the pathological features of spinal cord trauma such as tissue recovery, neuroinflammation, and Ca^2+^ imbalance by employing a surgical animal model of subacute SCIs.

Following an SCI, the partial or total loss of the sensorimotor capacity results in paraplegia or quadriplegia [26]. Both conditions notably trouble the well-being of injured subjects, limiting daily activities and requiring assistance for personal care [27].

As a consequence of the spinal cord traumatic event, our study showed a substantial decrease in the BMS score; nevertheless, SUN11602 oral administration significantly counteracted neurobehavioral impairment caused by an SCI after only one day of treatment and more consistently in a time-dependent manner until 10 days of treatment.

It has been extensively studied that motor deficits and loss of sensory inputs are principally linked to mechanical trauma, which, in addition to causing the local deformation of the spinal column, prompts the loss of motoneurons and the destruction of neuronal circuits [28]. Indeed, after mechanical damage occurs, the compression of the spinal cord leads to consequent neuronal degeneration, demyelination, blood vessel damage, and in situ neutrophil accumulation [29,30].

In this work, we clearly demonstrated that treatment with SUN11602 exerted a protective effect, as evidenced by the reduction in spinal cord damage, lower neutrophil infiltration, and the restoration of myelinated neurons.

It has been shown that some of the key cells that sustain the inflammatory process during the traumatic event are mast cells [31]. Indeed, these types of immune cells can interact with different CNS components, causing the release of several proinflammatory mediators into the surrounding neuronal environment, thus supporting neuroinflammation [31]. In accordance, we observed a substantial increase in mast cell hyperactivity following an SCI; however, three days of the administration of SUN11602 was highly effective in reducing mast cell infiltration.

In response to tissue injury and the consequent induction of inflammatory reactions, the natural homeostatic mechanisms found in the mast cell–glia network can be affected [32]. In fact, it is widely known that microglia also react to pro-inflammatory signals emitted by other non-neuronal cells like mast cells [32]. In response to this stimulus, activated glia prompt the development of an ongoing neuroinflammatory state that supports the processes of neuronal degeneration, resulting in a substantial upsurge of GFAP and IBA-1, which are considered trustworthy hallmarks of a brain injury.

In our study, we detected a significant increase in GFAP and IBA-1 expression in spinal cord tissues from SCI mice; by contrast, SUN11602 oral administration attenuated reactive astrocytes and microglia, confirming its ability to decrease inflammation in glial cells.

As stated, in the complex clinical setting of SCIs, the inflammatory cascade is a key pathophysiological element [33]. Through the activation of many pathways, inflammation promotes the development of the illness and exacerbates secondary damage [34]. Regarding this, the transcriptional factor NF-κB is essential in coordinating the cellular processes that underlie inflammation through the promotion and release of many pro-inflammatory mediators, including enzymes, cytokines, and chemokines [35]. Therefore, the development of potent anti-inflammatory therapies, aimed at modulating NF-κB activity and its biological interplays, could constitute a fascinating therapeutic approach in the preservation of neuronal integrity. In our study, the data obtained confirmed the establishment of the neuroinflammatory state led by the activation of the NF-κB factor. However, SUN11602 treatment had the ability to modulate the NF-κB pathway, thus considerably decreasing the expression of the proinflammatory enzymes iNOS and COX-2. Interestingly, a recent article by He et al. stated that the activation of the PI3K/AKT may reduce the inflammatory phenotype and regulate cellular survival after a traumatic spinal cord injury by blocking the NF-kB pathway [11]. Therefore, assuming PI3K/AKT signaling has an important role in controlling the inflammatory response in the subacute phase of SCI secondary injury, we assessed the effect of SUN11602 on this pathway. Our results clearly revealed that an SCI impaired the PI3K/AKT signaling pathway; conversely, SUN11602 acted as a positive regulator of the PI3K/AKT axis, demonstrating potent anti-inflammatory activities and neuronal protection through its re-establishment. In addition, discoveries in the literature validated a considerable activation of the p38 MAPK signaling pathway [36,37], which, during a traumatic event like an SCI, largely contributes to neuropathic pain and neurotoxicity [38]. Accordingly, following SUN11602 administration, our data indicated a consistent attenuation of p38 MAPK signaling cascades, confirming the good management of the neuroinflammatory state by this bFGF mimetic.

The reciprocal relationships between Ca^2+^ dyshomeostasis and neuroinflammatory signaling pathways have been extensively studied [39]. In fact, a rise in pro-inflammatory species compromises the control of Ca^2+^-regulating systems, thereafter increasing neuronal vulnerability through elevated levels of intracellular Ca^2+^ [39]. We have previously shown that overexpressing Calbindin-D-(28 k), a direct target of SUN11602, can regulate the buffer of Ca^2+^ signals, preventing calcium-mediated Calpain activation and rebalancing Ca^2+^ excess in neurons [18].

Thus, considering Ca^2+^ imbalance a feature of traumatic damage, we looked into SUN11602’s potential to increase Calbindin-D-(28 k) levels in neurons as an effective strategy for replacing a Ca^2+^ physiological condition. Our findings demonstrated that SUN11602 treatment increased the expression of Calbindin-D-(28 k), lowered the levels of S-100β, and inhibited Calpain activity. Hence, also in this pathological setting, SUN11602 treatment revealed an excellent control of intracellular Ca^2+^ efflux via Ca^2+^-binding protein modulation.

Many reports suggested considerable interplay between neurotrophins and Ca^2+^uptake [40,41,42]. In fact, it was demonstrated that prolonged neurotrophin expressions enhanced the expression of the Ca^2+^ regulatory proteins, promoting sprouting, synaptic rearrangement, and neuronal regeneration [40,41,42].

Considering these assumptions, the delivery of neurotrophic factors such as BDNF, GDNF, and NT-3 could be a promising field of investigation for spinal cord trauma as well [43]. Nevertheless, although endogenous neurotrophic factor levels do surge at various times following spinal cord lesions as part of the physiological response to nerve damage, their usefulness is constrained by their brief half-lives and poor blood–brain barrier permeability [44].

To solve this issue, CREB targeting, one of the key neurotrophin regulators, has been proven successful [45]. Indeed, CREB phosphorylation guarantees the restoration of the depleted endogenous neurotrophic factors, providing neuroprotection and regeneration activity against CNS disorders [46]. In this study, SUN11602-treated mice revealed a considerable upregulation of p-CREB expression and, in turn, of BDNF, GDNF, and NT-3 levels, thus supporting the neuroprotective capabilities of this bFGF mimetic in promoting neurotrophins upstream after spinal cord trauma.

## 4. Materials and Methods

### 4.1. Materials

SUN11602 was purchased by Tocris Bioscience (Bristol, UK). Unless otherwise indicated, all compounds were obtained from Sigma-Aldrich Company Ltd. (Milan, Italy). All other chemicals were of the highest commercial grade available. All stock solutions were prepared in non-pyrogenic saline (0.9% NaCl; Baxter, Italy, UK). SUN11602 was dissolved in 10% dimethyl sulfoxide (DMSO) and diluted in a saline solution.

### 4.2. Animals

Male adult CD1 mice, 6–8 weeks old (25–30 g, Envigo, Udine, Italy), were housed in a controlled environment and provided with standard rodent chow and water in stainless steel cages in a room kept at 22 ± 1 °C with a 12 h light and 12 h dark cycle. This study was performed following Italian regulations on the use of animals (D.M.116192) and Directive legislation (EU) (2010/63/EU) amended by Regulation (EU) 2019/1010 as well as ARRIVE guidelines. Moreover, the study was approved by the OPBA of Messina with the authorization number 537/2018-PR.

### 4.3. SCI Surgical Procedure

Mice were anesthetized intraperitoneally with xylazine and ketamine (0.16 and 2.6 mg/kg body weight, respectively). The SCI model was induced via longitudinal incision made in the midline of the back of the mice, exposing the paravertebral muscles and dissecting them away, exposing the T5 to T8 vertebrae [22]. The SCI was produced via extradural compression of the spinal cord at vertebrae T5 to T8 using an aneurysm clip with a closing force of 24 g for 1 min. During recovery from anesthesia, the mice were placed on a warm heating pad and covered with a warm towel. The animals were euthanized, and spinal cord tissues were collected for histological examinations and biochemical analyses three days after the SCI procedure. The control mice were only subjected to a laminectomy. Moreover, SUN11602 oral treatments at the doses of 1, 2.5, and 5 mg/kg were performed once daily for three days.

### 4.4. Experimental Groups

The mice were randomly divided as follows:

Group 1: Sham+Vehicle: the mice were subjected to a laminectomy, but the aneurysm clip was not applied; these mice were orally administered the vehicle solution once daily for three days after the laminectomy (N = 10).

Group 2: Sham+SUN11602 1 mg/kg: the mice were subjected to a laminectomy, but the aneurysm clip was not applied; these mice were orally administered 1 mg/kg of SUN11602 once daily for three days after the laminectomy (N = 10).

Group 3: Sham+SUN11602 2.5 mg/kg: the mice were subjected to a laminectomy, but the aneurysm clip was not applied; these mice were orally administered 2.5 mg/kg of SUN11602 once daily for three days after the laminectomy (N = 10).

Group 4: Sham+SUN11602 5 mg/kg: the mice were subjected to a laminectomy, but the aneurysm clip was not applied; these mice were orally administered 5 mg/kg of SUN11602 once daily for three days after the laminectomy (N = 10).

Group 5: SCI+Vehicle: the mice were subjected to an SCI plus an oral administration of the vehicle solution once daily for three days after the SCI (N = 10).

Group 6: SCI+SUN11602 1 mg/kg: the mice were subjected to an SCI plus an oral administration of 1 mg/kg of SUN11602 once daily for three days after the SCI (N = 10).

Group 7: SCI+SUN11602 2.5 mg/kg: the mice were subjected to an SCI plus an oral administration of 2.5 mg/kg of SUN11602 once daily for three days after the SCI (N = 10).

Group 8: SCI+SUN11602 5 mg/kg: the mice were subjected to an SCI plus an oral administration of 5 mg/kg of SUN11602 once daily for three days after the SCI (N = 10).

The doses (1, 2.5, and 5 mg/kg) and the route of administration of SUN11602 used in this study were based on the literature and our previous in vivo study [18]. Since we found no evidence of toxicity or improvement in spinal cord tissue compared with the Sham+vehicle group, the experimental data pertaining to the Sham groups treated with SUN11602 were solely reported in the behavioral test (BMS open-field score).

### 4.5. BMS Open-Field Score

In another experimental set, locomotor performance was analyzed using BMS for 10 days after injury [47]. The BMS scale ranges from 0 (complete paralyses) to 9 (normal hind limb function) and rates locomotion on such aspects of hind limb function as weight support, stepping ability, coordination, and toe clearance.

### 4.6. Histological Evaluation

Three days after the SCI, spinal cord tissue was collected and fixed in 10% (*w*/*v*) PBS-buffered formaldehyde solution at 25 °C for 24 h, dehydrated with graduated ethanol, and embedded in paraffin. Then, the obtained 7 μm thick sections were stained with hematoxylin and eosin (H&E) to investigate tissue morphology. The histological score followed a five-point scale on the basis of the following morphological criteria: (0) no pathological abnormalities; (1) small, scattered areas of axonal swelling, morphologically unremarkable tissue in >75% of the perilesional area; (2) significant damage with normal gross architecture, unremarkable tissue in 50–75% of the perilesional area; (3) significant damage with normal gross architecture, morphologically unremarkable tissue in 25–50% of the perilesional area; (4) significant damage and loss of gross architecture in large areas, morphologically unremarkable tissue in 10–25% of the perilesional area; (5) complete dissolution of the spinal cord tissue over the entire perilesional area with loss of gross architecture, morphologically unremarkable tissue in <10% of the perilesional area. The results from every section of the spinal cord were averaged to obtain a final score (1 to 5) for each mouse. The results of the histological examinations were acquired using a Nikon Eclipse Ci-L microscope and showed at 20× (50 µm scale bar) and 40× magnifications (20 µm scale bar). Histological studies were performed in a blinded fashion by experienced histopathologists.

### 4.7. Luxol Fast Blue (LFB) Staining

To assess the degree of myelination/demyelination, staining with the Luxol Fast Blue (LFB) stain kit (Abcam, Waltham, MA 02453, USA # ab150675) was performed as briefly described below. Sections were deparaffinized and incubated in LFB solution at 56 °C O/N, then washed in 95% alcohol. Subsequently, the sections were incubated in lithium carbonate solution and 70% ethyl alcohol and finally counterstained in the cresyl violet solution. After dehydration, the sections were assembled with Eukitt (Bio-Optica, Milan, Italy) and observed by a light microscopy Eclipse Ci-L microscope. The slides were analyzed by a pathologist blinded to the treatment groups. Images were taken focusing on the perilesional area of the SCI and shown at 20× and 40× magnification.

### 4.8. Toluidine Blue Staining

Toluidine blue staining was performed according to a previously described method [48]. Sections were deparaffinized in xylene and dehydrated via graded successions of ethanol, 5 min in each solution. The sections were next sited in water for 5 min, relocated to toluidine blue for 4 min, and then cautiously blotted. Sections were positioned in absolute alcohol for 1 min, cleared in xylene, and fixed on glass slides using Eukitt (Bio-Optica, Milan, Italy). The number of stained mast cells was obtained by counting five high-power fields (40×) per section using a Nikon Eclipse Ci-L microscope. Images are shown at 40× magnification.

### 4.9. The Immunolocalization of NT-3 in Spinal Cord Tissues

Immunohistochemical localization was performed as previously described [49] and briefly reported. Specifically, sagittal spinal cord sections were deparaffinized and rehydrated as previously described. Then, the sections were incubated overnight (O/N) with primary mouse NT-3 (1:100; sc-518099; Santa Cruz Biotechnology). Sections were washed with PBS and incubated with peroxidase-conjugated bovine anti-mouse immunoglobulin G (IgG) secondary antibody (1:2000 Jackson Immuno Research, West Grove, PA, USA). Immunohistochemical images were obtained and observed using a Nikon Eclipse Ci-L microscope. Immunoreactivity (brown staining) was determined by counting the number of positive cells at 40× magnification within five random fields. The analysis was performed using ImageJ. For each mouse in the different experimental groups, we reported the mean of positive cells detected. The histogram as well as statistical analysis was executed with GraphPad version 8.0 (La Jolla, CA, USA). Analyses were performed blindly. Images are shown at 20× and 40× magnification.

### 4.10. The Immunofluorescence Staining of GFAP and IBA-1 in Spinal Cord Tissues

Spinal cord sections were processed for immunofluorescence staining as previously reported [50]. Sections were incubated with anti-glial fibrillary acidic protein (anti-GFAP) (1:100, sc-9065, Santa Cruz Biotechnology), or anti-ionized calcium binding adaptor molecule 1 (anti-IBA-1) (1:100, sc-32725, Santa Cruz Biotechnology) antibodies in a humidified chamber O/N at 37 °C.

After 24 h of incubation, the spinal cord sections were washed with PBS and then incubated with conjugated antirabbit Alexa Fluor-488 secondary antibody #A32731 (1:1000 in PBS, *v/v* Molecular Probes, Monza, Italy) for 3 h at room temperature.

Nuclei were stained by adding 2 μg/mL 4′,6′-diamidino-2-phenylindole (DAPI; Hoechst, Frankfurt, Germany) in PBS. Sections were observed at 40× magnification using a Nikon Eclipse Ci-L microscope. Contrast and brightness were established by examining the most brightly labeled pixels and applying settings that allowed for the clear visualization of structural details while keeping the highest pixel intensities close to 250. The same settings were used for all images obtained from the other samples that had been processed in parallel. Images are shown at 40× magnification.

### 4.11. The Western Blot Analysis of BDNF, GDNF, IkBa, NF-kB, PI3K, p-p38, p-AKT, p-CREB, Calbindin-D28K, and Anti-S100b in Spinal Cord Tissues

Nuclear and cytosolic extracts were prepared as previously mentioned [51]. Briefly, spinal cord protein samples were heated at 95 °C for 5 min and subsequently loaded onto SDS-PAGE gel (percentage was chosen based on the molecular weight of the investigated protein, usually 10 or 12%). Electrophoresis was started at 70 V and was then increased to 100 V until the end of the electrophoretic run for approximately 2 h.

Thereafter, proteins were transferred to a polyvinylidene difluoride (PVDF) membrane; after this procedure, the membranes were blocked with 5% (*w*/*v*) nonfat dried milk in buffered saline (PM) for 1 h at room temperature.

Anti-pro-BDNF (1:500; sc-65514; Santa Cruz Biotechnology), anti-GDNF (1:500; sc-328; Santa Cruz Biotechnology) for neurotrophic factors, anti-NF-κB (1:500; sc-8008; Santa Cruz Biotechnology), anti-IκB-a (1:500; sc-1643: Santa Cruz Biotechnology), anti-COX-2 (1:500; sc-376861; Santa Cruz Biotechnology), anti-iNOS (1:500; sc-7271; Santa Cruz Biotechnology), anti-PI3K (1:2000; ElabScience), anti-p-p38 (1:500; sc-166182; Santa Cruz Biotechnology), anti-p-AKT (1:500; sc-7985-R; Santa Cruz Biotechnology), anti-p-CREB (1:500; sc-81486; Santa Cruz Biotechnology), anti-Calbindin-D28K (1:500; sc-365360; Santa Cruz Biotechnology), anti-S100b (1:500; sc-393919; Santa Cruz Biotechnology), anti-β-actin (1:500; sc-8432; Santa Cruz Biotechnology), anti-AKT (1:500; sc-5298; Santa Cruz Biotechnology), anti-p38 (1:500; sc-81621; Santa Cruz Biotechnology) and anti-CREB (1:500; sc-374227; Santa Cru biotechnology) for cytosolic fraction and anti-Lamin A/C (1:1000; sc-376248; Santa Cruz biotechnology) for nuclear fraction were used. Primary antibodies were incubated overnight. Finally, membranes were incubated for 1 h at room temperature with a secondary anti-mouse antibody (1:1000, Jackson ImmunoResearch Laboratories; West Grove, PA, USA; cat#115-035-068;) or a secondary anti-rabbit antibody (1:1000, Jackson ImmunoResearch Laboratories; West Grove, PA, USA, cat#111-035-003). The bands were obtained using a chemiluminescence detection system (ECL) according to the manufacturer’s instructions (Thermo, Waltham, MA, USA). Then, images of blot signals were imported to analysis software ImageJ (1.52k) and standardized to Lamin A/C or β-actin and expressed as a percentage of the control.

### 4.12. Calpain Activity

Calpain activity was assessed fluorometrically according to previous studies [52,53]. Briefly, the spinal cord samples were homogenized and centrifuged, and the obtained supernatant was subjected to Ca-dependent fluorescence and non-Ca-dependent fluorescence to determine calpain activity, using N-succinyl-Leu-Tyr-(N-succinyl-LY)-AMC, cleaved by μ/m-calpain. Finally, samples were incubated in Buffer A, containing 63 mm imidazole–HCl, pH 7.3, 10 mm B-mercaptoethanol, and 5 mm CaCl_2_; then they were cleaved with 150 μm M-succinyl-LY-AMC to measure Ca-dependent fluorescence. In the same way, to measure non-Ca-dependent fluorescence, we used Buffer A without calcium containing 1 mm EDTA and 10 mm EGTA.

### 4.13. MPO Assay

To evaluate the presence of infiltrating cells, specifically neutrophils, an MPO assay was performed according to previous studies [48]. Briefly, the spinal cord samples were homogenized in a solution containing 0.5% (*w/v*) hexadecyltrimethyl ammonium bromide dissolved in 10 mM potassium phosphate buffer (pH 7) and centrifuged at 20,000× *g* at 4 °C for 30 min. An aliquot of the supernatant was then allowed to react with a solution of 1.6 mM tetramethylbenzidine and 0.1 mM H_2_O_2_. The absorbance of the supernatant was measured via spectrophotometry at 650 nm. MPO activity was expressed as units of MPO/mg of proteins.

### 4.14. Statistical Evaluation

All values are expressed as mean ± SD. The results were analyzed using a One-Way or Two-Way analysis of variance followed by a Bonferroni post hoc test for multiple comparisons. A *p*-value of less than 0.05 was considered significant.

## 5. Conclusions

In summary, the findings from this study highlighted the advantages of administering SUN11602, proving for the first time that this bFGF mimetic has neuroprotective properties following spinal cord injuries. Together, these beneficial outcomes led to a better management of SCI features, a decrease in neurobehavioral impairment and neuroinflammation, and an improvement in Ca^2+^ disruption as well as neurotrophins in the subacute phase of a spinal cord traumatic event. Taking into account these new discoveries, SUN11602 may be a promising therapeutic strategy to promote tissue regeneration and the subsequent survival of neurons, representing a valuable means of support in the pharmacological approach for injured patients. Nevertheless, considering animal model limits, future in-depth clinical research is required to corroborate these early findings in order to expand our knowledge of SCI patient care and the pharmaco-toxicological aspects of the SUN11602 compound.

## Data Availability

All data in this study are included in this published article.

## Abbreviations

SCI	Spinal cord injury
GFAP	Glial fibrillary acidic protein
IBA-1	ionized calcium binding adaptor molecule 1
TNF-α	Tumor necrosis factor alpha
IL-1β	Interleukin-1 beta
IFN-γ	Interferon–gamma
S100-β	S100 calcium-binding protein B
CNS	Central Nervous System
bFGF	Basic fibroblast growth factor
NF-κB	Nuclear factor kappa B
WHO	World Health Organization
FGFRs	Fibroblast growth factor receptors
IκB-α	Nuclear factor of kappa light polypeptide gene enhancer in B-cells inhibitor, alpha
Calb1	Calbindin-D28k
PD	Parkinson disease
MPO	Myeloperoxidase
BMS	Basso Mouse Scale
H&E	Hematoxylin and eosin
LFB	Luxol Fast Blue
MCs	Mast Cells
PI3K	Phosphoinositide 3-kinase
AKT	AKT serine/threonine kinase 1/Protein kinase B
MAPK	Mitogen-activated protein kinases
COX-2	Cyclooxygenase 2
iNOS	Inducible nitric oxide synthase
p38	p38 mitogen-activated protein kinases
PKCδ	Protein kinase C delta type
ELISA	enzyme-linked immunosorbent assay
BDNF	Brain-derived neurotrophic factor
GDNF	Glial cell line-derived neurotrophic facto
NT-3	Neurotrophin-3
CREB	cAMP response element-binding protein
DMSO	Dimethyl sulfoxide
PBS	Phosphate-buffered saline
IgG	Immunoglobulin G
DAPI	4′,6-diamidino-2-phenylindole
SDS-PAGE	Sodium dodecyl sulfate–polyacrylamide gel electrophoresis
PVDF	Polyvinylidene difluoride
